# Efficacy of Respiratory Muscle Training in the Immediate Postoperative Period of Cardiac Surgery: A Systematic Review and Meta-Analysis

**DOI:** 10.21470/1678-9741-2022-0165

**Published:** 2024-01-30

**Authors:** Tarcísio Nema de Aquino, João Paulo Prado, Ernesto Crisafulli, Enrico Maria Clini, Giovane Galdino

**Affiliations:** 1 Instituto de Ciências da Motricidade, Universidade Federal de Alfenas, Alfenas, Minas Gerais, Brazil; 2 Department of Rehabilitation and Cardiology, Hospital Santa Lúcia, Poços de Caldas, Minas Gerais, Brazil; 3 Department of Medicine and Surgery, Respiratory Disease and Lung Function Unit, University of Parma, Parma, Italy; 4 Department of Medical and Surgical Sciences, University of Modena and Reggio Emilia and University Hospital of Modena Policlinico, Modena, Italy

**Keywords:** Cardiac Surgery, Respiratory Muscle Training, Functional Capacity, Pulmonary Function, Length of Hospital Stay, Meta-Analysis, Systematic Review

## Abstract

**Introduction:**

This study aimed to evaluate the efficacy of respiratory muscle training during the immediate postoperative period of cardiac surgery on respiratory muscle strength, pulmonary function, functional capacity, and length of hospital stay.

**Methods:**

This is a systematic review and meta-analysis. A comprehensive search on PubMed®, Excerpta Medica Database (or Embase), Cumulative Index of Nursing and Allied Health Literature (or CINAHL), Latin American and Caribbean Health Sciences Literature (or LILACS), Scientific Electronic Library Online (or SciELO), Physiotherapy Evidence Database (or PEDro), and Cochrane Central Register of Controlled Trials databases was performed. A combination of free-text words and indexed terms referring to cardiac surgery, coronary artery bypass grafting, respiratory muscle training, and clinical trials was used. A total of 792 studies were identified; after careful selection, six studies were evaluated.

**Results:**

The studies found significant improvement after inspiratory muscle training (IMT) (n = 165, 95% confidence interval [CI] 9.68, 21.99) and expiratory muscle training (EMT) (n = 135, 95% CI 8.59, 27.07) of maximal inspiratory pressure and maximal expiratory pressure, respectively. Also, IMT increased significantly (95% CI 19.59, 349.82, n = 85) the tidal volume. However, no differences were found in the peak expiratory flow, functional capacity, and length of hospital stay after EMT and IMT.

**Conclusion:**

IMT and EMT demonstrated efficacy in improving respiratory muscle strength during the immediate postoperative period of cardiac surgery. There was no evidence indicating the efficacy of IMT for pulmonary function and length of hospital stay and the efficacy of EMT for functional capacity.

## INTRODUCTION

**Table:** 

Abbreviations, Acronyms & Symbols				
6MWT	= Six-minute walk test		IMS	= Inspiratory muscle strength
CABG	= Coronary artery bypass grafting		IMT	= Inspiratory muscle training
CG	= Control group		LILACS	= Latin American and Caribbean Health Sciences Literature
CI	= Confidence interval		MD	= Mean difference
CINAHL	= Cumulative Index of Nursing and Allied Health Literature		MEP	= Maximal expiratory pressure
CVD	= Cardiovascular disease		MIP	= Maximal inspiratory pressure
Embase	= Excerpta Medica Database		PEDro	= Physiotherapy Evidence Database
EMS	= Expiratory muscle strength		PO3	= Third postoperative day
EMT	= Expiratory muscle training		RMT	= Respiratory muscle training
EPAP	= Expiratory positive airway pressure		SciELO	= Scientific Electronic Library Online
EPF	= Expiratory peak flow		SD	= Standard deviation
GRADE	= Grading of Recommendations, Assessment, Development and Evaluation		TV VC	= Tidal volume = Vital capacity
IG	= Intervention group			

Approximately 1.9 million people worldwide die each year from cardiovascular diseases (CVDs)^[1]^. In order to control these diseases, significant advances have been made in clinical practice for both diagnostic and treatment purposes^[2]^. Although conservative treatment may reduce CVD mortality rate, surgical treatment is necessary in many cases. Thereby, coronary artery bypass grafting (CABG) and valve replacement have been the gold standard treatments^[2,3]^. However, cardiac surgery may lead to several complications, mainly induced by sternotomy and extracorporeal circulation, such as respiratory muscle weakness, reduction in pulmonary function, and pulmonary infections, especially in the immediate postoperative period, which comprises the first hours after surgery until hospital discharge^[4,5]^. Furthermore, in order to avoid sternotomy-induced pain, patients maintain a shallow breathing pattern, which would restrict their chest movement, leading to a loss of respiratory muscle strength and diaphragm dysfunction^[6]^. Taken together, these cardiac surgery-induced changes increase the risk of mortality, length of stay, and patient costs^[6]^.

In this scenario, the role of physiotherapy is extremely important. Physiotherapy includes a range of techniques, such as early mobilization, breathing exercises, coughing techniques, incentive spirometry, continuous positive airway pressure, and respiratory muscle training (RMT), in the preoperative and postoperative periods of cardiac surgery, in order to prevent complications^[7,8]^. Moreover, especially in the immediate postoperative period, this intervention is essential.

RMT is widely used and recognized among the physiotherapeutic approaches used during the pre and postoperative periods of cardiac surgery. It increases inspiratory and expiratory muscle strength, as well as preventing respiratory muscle weakness, and reducing respiratory complications^[9,10]^. Several devices can be used for respiratory muscle strength training, for example, IMT Respironics Threshold™ (Philips, Philadelphia, United States of America) and DHD IMT (DHD Medical Products, New York, United States of America) for inspiratory muscle training (IMT), and Expiratory Positive Airway Pressure (Ventus Medical, California, United States of America) and Respilift (Medinet, Italy) for expiratory muscle training (EMT)^[9-11]^.

Although several studies have demonstrated the importance of respiratory strength training after cardiac surgery, few studies have evaluated this treatment in the immediate postoperative period, and most of them used inspiratory strength training^[12-15]^. A meta-analysis had already shown the benefits of IMT (improvement of inspiratory muscle strength, pulmonary function, and functional capacity) before and after cardiac surgery^[11]^, however, without investigating the effect of expiratory strength training. In addition, to our knowledge, no study has evaluated the effect of RMT in the immediate postoperative period of cardiac surgery based on the overall quality of evidence of the Grading of Recommendations, Assessment, Development and Evaluation (GRADE) approach^[16]^, which offers a transparent structure for presenting evidence with recommendations for clinical practice.

This systematic review and meta-analysis aimed to investigate and evaluate the effects of IMT and EMT on respiratory muscle strength, pulmonary function, functional capacity, and hospital stay in the immediate postoperative period of cardiac surgery in comparison to usual care, based on the quality of evidence by the GRADE approach.

## METHODS

### Protocol and Registration

This meta-analysis was previously registered in the International Prospective Register of Systematic Reviews - PROSPERO (registration number CRD42018092593), following the Preferred Reporting Items for Systematic Reviews and Meta-Analysis (or PRISMA) guidelines^[17]^.

### Eligibility Criteria

The present study included randomized controlled trials that evaluated the efficacy of EMT and/or IMT in patients after the immediate postoperative period of cardiac surgery, except heart transplantation, before hospital discharge. Studies that (I) performed RMT before cardiac surgery and (II) which the sample size was not provided by the authors were excluded.

### Types of Interventions

The studies evaluated used mainly groups of patients undergoing inspiratory strength training, IMT, and EMT compared to groups of patients undergoing usual care. RMT was conducted using devices with inspiratory or expiratory loads, while the control group was represented by usual care, such as early mobilization, bronchial hygiene, breathing exercises without loads, and visits of nursing and medical staff.

### Outcomes

The main outcomes considered in this review were respiratory muscle strength, evaluated by maximal inspiratory and expiratory pressures (cmH₂O); lung function, evaluated by tidal volume (mL) and expiratory peak flow (L/min); and functional capacity, evaluated by six-minute walk test (m) and length of hospital stay (days). All outcomes were analyzed within a period of up to 10 days of hospitalization since after that period, the patients had already been discharged from the hospital.

### Electronic Search

In this study, PubMed®, Excerpta Medica Database (or Embase), Cumulative Index of Nursing and Allied Health Literature (or CINAHL), Latin American and Caribbean Health Sciences Literature (or LILACS), Scientific Electronic Library Online (or SciELO), Physiotherapy Evidence Database (PEDro), and Cochrane Central Register of Controlled Trials databases were systematically searched as of March 2019. The search terms were based on the strategies suggested in the Cochrane Handbook for Systematic Reviews of Interventions, and the searches were adjusted for each database. A combination of free-text words and indexed terms referring to cardiac surgery, CABG, RMT, and clinical trials were used.

The reference lists from previous systematic reviews and clinical trials eligible for this review were also examined. We searched for ongoing clinical trials on ClinicalsTrials.gov, the International Standard Randomised Controlled Trial Number (or ISRCTN) Registry, the Australian New Zealand Clinical Trials Registry (or ANZCTR), and the International Clinical Trials Registry Platform (or ICTRP), up to March 2019. There were no restrictions regarding the language and publication dates of the potentially eligible studies.

### Study Selection

The study selection was performed by two independent coauthors (T.N.A. and G.G.) in two phases to determine which articles were suitable. At first, duplicated and nonrelevant studies were discarded by examining titles and abstracts. Secondly, in accordance with the study inclusion and exclusion criteria, eligible studies were extracted by reviewing full-text articles. Any disagreements between the reviewers were resolved by consensus, and if necessary, a third reviewer (J.P.P.) was asked to decide on the inclusion of the studies.

### Data Collection Process

The data relating to the number of participants and their characteristics (sex, age, and type of cardiac surgery) were extracted. A description of the intervention and the comparisons, the outcome assessment tools, and the study results were also obtained. Lastly, for studies in which some data was not presented in the manuscript, we contacted the authors^[18]^, and the data were provided successfully.

### Assessment of Methodological Quality of Studies

To assess the methodological quality of the studies, we use the PEDro scale^[19]^, which is an 11-item scale designed for rating methodological quality of clinical trials. Each satisfied item (except for item 1, which, unlike other scale items, pertains to external validity) contributes one point to the total PEDro scale (range = 0-10 points). The PEDro scores ranged from 4 to 10 - scores ranging from 9 to 10 were considered methodologically to be of “excellent” quality, scores from 6 to 8 were of “good” quality, scores 4 or 5 were of “fair” quality, and scores < 4 were felt to be of “poor” quality. Of all six studies evaluated using the PEDro scale, only one study was of “high quality”^[18]^. In addition to the PEDro score and according to the GRADE approach, when > 25% of the studies were of low quality, the assessment of the quality of the evidence was downgraded due to the risk of bias^[16]^.

### Quality Assessment of the Evidence

The overall quality of the evidence of the studies was rated in accordance with the GRADE approach^[16]^. It consists of five items: (I) study limitations (risk of bias); (II) inconsistency of results (heterogeneity); (III) indirectness of evidence; (IV) imprecision of the effect estimates; and (V) reporting bias. The quality of the evidence was classified into four categories: high, moderate, low, and very low^[20]^. This approach entails the downgrading of evidence from high to moderate, to low, and to very low quality based on certain criteria. The criteria for downgrading the evidence one level were: (I) for study limitation, if the majority of studies (> 50%) was rated as high risk of bias; (II) for inconsistency, if heterogeneity was greater than the accepted low level (*I^2^* > 40%); (III) for indirectness, if the RMT session does not correspond to what is used in clinical practice; and (IV) for imprecision, if meta-analysis had small sample size (n < 300).

### Statistical Analysis

All analyses were accomplished by random-effects models^[21]^. Median and standard deviation were used as summary statistics in meta-analysis once outcome measurements in all studies had the same scale^[22]^. In addition, the heterogeneity of results across the studies was evaluated using the *I^2^* statistic, interpreted as might not be important (0%-40%), may represent moderate (30%-60%), may represent substantial (50%-90%), and considerable (75%-100%) heterogeneity^[22]^. For meta-analysis, the Review Manager Software version 5.3 (RevMan, Copenhagen, Denmark) was used, which provides combined estimates with a 95% confidence interval (CI).

## RESULTS

### Study Selection

**Fig. 1:**
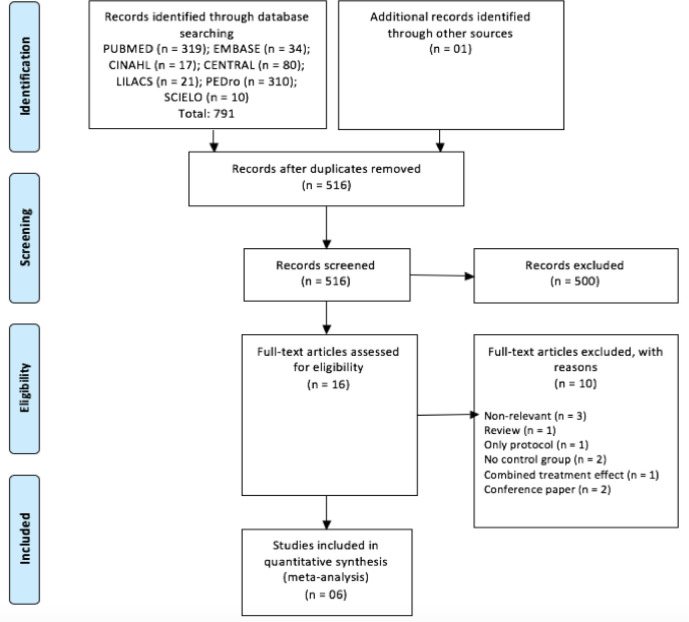
Flowchart for trial selection according to Preferred Reporting Items for Systematic Reviews and Meta-analysis (or PRISMA). CINAHL=Cumulative Index of Nursing and Allied Health Literature; Embase=Excerpta Medica Database; LILACS=Latin American and Caribbean Health Sciences Literature; PEDro=Physiotherapy Evidence Database; SciELO=Scientific Electronic Library Online.

A total of 792 studies were identified in the literature search process to seek out systematic reviews with meta-analysis focused on this field. A consensus was reached and ended in a total of six potentially relevant studies, which were reviewed in full text, met the eligibility criteria, and were included in this review^[12-15,23]^ (Figure 1). The studies were published between 2009 and 2018, with a total of 298 participants. In addition, men had a higher frequency of CVDs (72%).

Of the six studies, four were conducted in Brazil^[13-15,23]^, one in India^[12]^, and one in Italy^[18]^. Regarding the effect of muscle strength training on outcomes, four trials assessed IMT^[12-15]^. The pulmonary function outcome, assessed by tidal volume and expiratory peak flow, was investigated by two studies^[14,15]^. In addition, two studies assessed also hospitalization days^[13,15]^, and two assessed EMT and functional capacity^[18,23]^ (Table 1). Importantly, all six of these selected studies assessed patients immediately after the intervention.

**Table 1:** Overview of study design, participants, intervention, and results.

Study	Participants			Protocol used		Results
	Male/female number	Cardiac surgery type	Average age ± SD and n. of patients per group	Intervention	Control	
Stein et al. (2009).	20 (11/9)	CABG	IG: 10 (64 ± 7)	EMT: EPAP with progressive resistance increase of 3 to 8 cmH₂0 for 3 to 12 minutes.	Nursing care, orientations once a day, but without respiratory exercises.	No significant differences were found in MIP in both groups after surgery. IG presented greater distance walked in the 6MWT compared to CG.
PEDro score: 5/10			CG: 10 (63 ± 6)			Conclusion: Although no difference was found compared to CG in the immediate postoperative period, training with EPAP proved to be easy to use for patients.
Praveen et al. (2009).	30 (missing data)	CABG	IG: 15 (57.2 ± 85.62)	IMT: 40% of the constant MIP in the Thresholdä IMT. Three sets of 10 repetitions a day for 7 days, supervised.	Bronchial hygiene maneuvers (vibrocompression), postural drainage, and tracheal aspiration when necessary.	There was a significant difference between groups regarding MIP, MEP, EPF, and TV. There were no differences in the length of stay, dyspnea, and pain.
PEDro score: 4/10			CG: 15 (55.6 ± 5.26)			Conclusion: IMT is effective in improving IMS and EMS after cardiac surgery.
Barros et al. (2010).	38 (29/9)	CABG	IG: 23 (62.13 ± 8.1)	IMT: 40% of the constant MIP in the Thresholdä IMT. Three sets of 10 repetitions a day for 7 days, supervised.	Bronchial hygiene maneuvers (vibrocompression), postural drainage, and tracheal aspiration when necessary.	Significant differences were found between groups regarding MIP, MEP, EPF, and TV. There were no differences in relation to the length of stay, dyspnea, and pain.
PEDro score: 4/10			CG: 15 (67.08 ± 7.11)			Conclusion: IMT is effective in improving IMS and EMS after cardiac surgery.
Matheus et al. (2012).	47 (34/13)	CABG	IG: 23 (61.83 ± 8.61)	IMT: 40% of the constant MIP in the Thresholdä IMT. Three sets of 10 repetitions twice a day for 3 days.	Preoperative orientations, pulmonary reexpansion with fractional patterns, respiratory incentive, orthostatism, and walking.	After cardiac surgery (PO3), IMT induced an improvement in VC and TV compared to CG.
PEDro score: 4/10			CG: 24 (66.33 ± 10.2)			Conclusion: IMT is effective in improving ventilation and lung function in the immediate postoperative period of cardiac surgery.
Crisafulli et al. (2013)	48 (37/11)	CABG, aortic and mitral valve replacement, mitral valve repair	IG: 24 (67.3 ± 7.8)	EMT: Respilift with a load of 30 cmH20. Fifteen minutes twice a day, supervised.	Respilift with a sham load (no resistance) for 15 minutes twice a day, supervised.	After the intervention, there was a significant increase in MEP (*P*<0.001).
PEDro score: 8/10			CG: 24 (67.5 ± 10.5)			Conclusion: Respilift is a feasible and effective device for EMT for use after cardiac surgery.
Cordeiro et al. (2016).	50 (27/23)	CABG, valve replacement surgery, congenital cardiac surgery	IG: 25 (56.4 ± 13)	IMT: 40% of the constant MIP in the Thresholdä IMT. Three sets of 10 repetitions twice a day until hospital discharge.	Patients were submitted to care routines not described by the authors.	A significant increase in the MIP (*P*<0.007) and 6MWT (*P*<0.003) was found after intervention (IG).
PEDro score: 3/10			CG: 25 (57 ± 14.7)			Conclusion: IMT is effective in improving functional capacity and IMS in the immediate postoperative period of cardiac surgery.

6MWT=six-minute walk test; CABG=coronary artery bypass grafting; CG=control group; EMS=expiratory muscle strength; EMT=expiratory muscle training; EPAP=expiratory positive airway pressure; EPF=expiratory peak flow; IG=intervention group; IMS=inspiratory muscle strength; IMT=inspiratory muscle training; MEP=maximal expiratory pressure; MIP=maximal inspiratory pressure; PEDro=Physiotherapy Evidence Database; PO3=third postoperative day; SD=standard deviation; TV=tidal volume; VC=vital capacity

For IMT interventions, the IMT Respironics Threshold™ or the DHD IMT devices with a load equivalent to 40% of the maximal inspiratory pressure were used. When the intervention was EMT, the PEP Respironics Threshold™ and Respilift devices were used. The control groups were characterized by usual care, such as placebo treatment, nursing care, deep breathing, and early mobilization.

### Methodological Quality of Studies

Five of the six studies had already identified the risk of bias from the PEDro database^[12-14,18]^. In accordance with the PEDro score, the mean for risk of bias of the six studies evaluated in this review was 4.6 (standard deviation = 1.75), which indicates that they are considered to be of fair quality (Table 2).

**Table 2:** Methodological quality of studies using the Physiotherapy Evidence Database (PEDro) score.

Criteria^*^												
	1	2	3	4	5	6	7	8	9	10	11	Total
Stein et al. (2009)	Y	Y	N	Y	N	N	Y	N	N	Y	Y	5
Praveen et al. (2009)	Y	Y	N	Y	N	N	N	N	N	Y	Y	4
Barros et al. (2010)	Y	Y	N	Y	N	N	N	N	N	Y	Y	4
Matheus et al. (2012)	Y	Y	N	Y	N	N	N	N	N	Y	Y	4
Crisafulli et al. (2013)	Y	Y	N	Y	Y	Y	N	Y	Y	Y	Y	8
Cordeiro et al. (2016)	Y	N	N	Y	N	N	N	N	N	Y	Y	3

* PEDro criteria: (1) specification of eligibility criteria (this criterion was not counted for the final score), (2) random allocation, (3) concealed allocation, (4) prognostic similarity at baseline, (5) participant blinding, (6) therapist blinding, (7) outcome assessor blinding, (8) > 85% follow-up of at least one key outcome, (9) intention to treat analysis, (10) between or within-group statistical comparison, (11) point estimates of variability provided

### Treatment Effects

#### Comparison Between Inspiratory Muscle Training and Usual Care for Inspiratory Muscle Strength

**Fig. 2:**
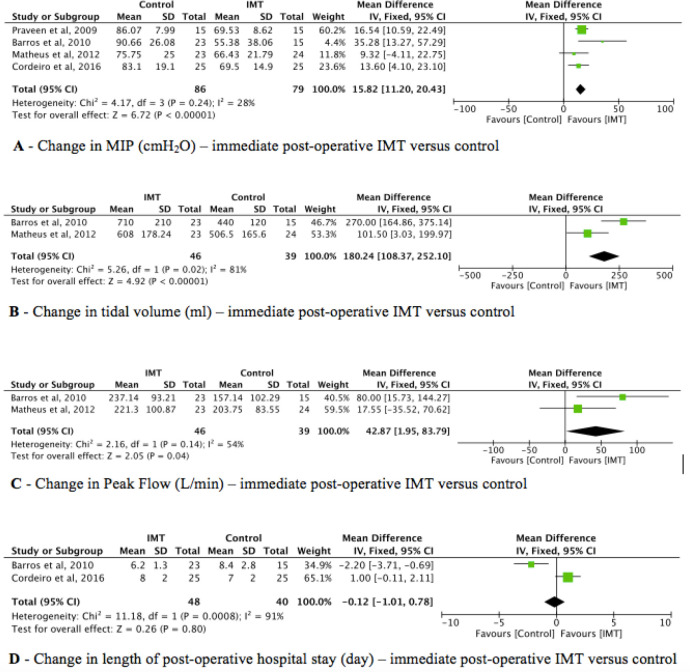
Comparison between respiratory muscle training and usual care on respiratory muscle strength, pulmonary function, length of hospital stay, and functional capacity. CI=confidence interval; IMT=inspiratory muscle training; MIP=maximal inspiratory pressure; SD=standard deviation.

Four included trials (n = 165) investigated the effect of IMT compared to usual care^[12-15]^. The pooled estimate showed that IMT improved inspiratory muscle strength by 15.83 cmH₂O (95% CI: 9.68-21.99, *I^2^* = 28%) (Figure 2A). According to the GRADE approach, the overall quality of evidence was rated as low quality (*i.e.*, downgraded for the risk of bias, imprecision, and publication bias) (Table 3).

**Table 3:** Evidence of inspiratory muscle training compared to control (usual care) for inspiratory muscle strength, pulmonary function, and length of hospital stay.

Certainty assessment							N. of patients		Effect	Certainty
N. of studies	Study design	Risk of bias	Inconsistency	Indirectness	Imprecision	Other considerations	Inspiratory muscle training	Conventional physiotherapy	Absolute (95% CI)	
Maximal inspiratory pressure (assessed with manovacuometry - cmH₂O)										
4	Randomized trials	Serious^a^	Not serious^b^	Not serious	Not serious	Publication bias strongly suspectedc	86	79	MD 15.82 cmH₂O higher (11.2 higher to 20.43 higher)	⨁⨁◯◯
										Low
Expiratory peak flow (assessed with spirometry - L/min)										
2	Randomized trials	Serious^a^	Serious^b^	Not serious^a^	Serious^d^	Publication bias strongly suspectedc	46	39	MD 42.87 L/min higher (1.95 higher to 83.79 higher)	⨁◯◯◯
										Very low
Tidal volume (assessed with spirometry - ml)										
2	Randomized trials	Serious^a^	Serious^b^	Not serious^a^	Serious^d^	Publication bias strongly suspectedc	46	39	MD 180.24 mL higher (108.37 higher to 252.1 higher)	⨁◯◯◯
										Very low
Length of hospital stay (days)										
2	Randomized trials	Serious^a^	Serious^b^	Not serious^a^	Serious^d^	Publication bias strongly suspectedc	48	40	MD 0.12 days lower (1.01 lower to 0.78 higher)	⨁◯◯◯
										Very low

CI=confidence interval; MD=mean difference

a All studies presented a PEDro score of 4 out of 10

b Based on statistical criteria (*I^2^*) and the small number of published studies

c There are studies registered, but not published

dBased on a small number of events, we consider reducing the of evidence, even with the seemingly narrow CI

#### Comparison Between Inspiratory Muscle Training and Usual Care for Tidal Volume

The investigation of IMT compared to usual care for tidal volume after cardiac surgery was performed by two clinical trials (n = 85)^[13,14]^. According to the pooled estimate, IMT improved the tidal volume by 184.7 mL (95% CI: 19.59-349.82, *I^2^* = 81%) (Figure 2B). The overall quality of evidence according to the GRADE approach was rated as very low quality (*i.e.*, downgraded for inconsistency, imprecision, risk of bias, and publication bias) (Table 3).

#### Comparison Between Inspiratory Muscle Training and Usual Care for Expiratory Peak Flow

To compare the effect of IMT to the effect of usual care on expiratory peak flow, two trials were included (n = 85)^[13,14]^. There was no difference between the studies about expiratory peak flow by pooled estimate (95% CI: -14.93-107, *I^2^* = 54%) (Figure 2C). Thus, the overall quality of evidence was rated as very low quality (*i.e.*, downgraded for inconsistency, imprecision, risk of bias, and publication bias), according to the GRADE approach (Table 3).

#### Comparison Between Inspiratory Muscle Training and Usual Care for the Length of Hospital Stay

Two included trials (n = 88) investigated the effect of IMT compared to usual care^[13,15]^. The pooled estimate showed that there was no difference between the studies about the length of hospital stay (95% CI: -3.69-2.58, *I^2^* = 91%) (Figure 2D). The overall quality of evidence according to the GRADE approach was rated as very low quality (*i.e.*, downgraded for inconsistency, imprecision, risk of bias, and publication bias) (Table 3).

#### Comparison Between Expiratory Muscle Training and Minimal Interventions for Expiratory Muscle Strength

**Fig. 3:**
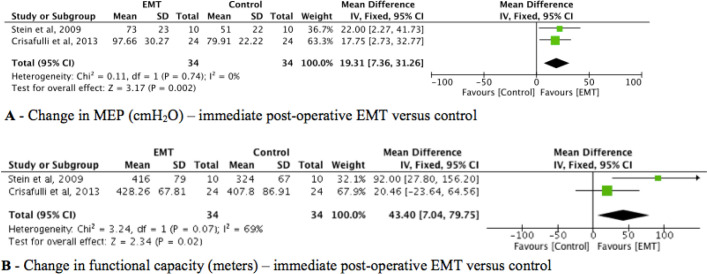
Evidence level of respiratory muscle training on respiratory muscle strength, pulmonary function, length of hospital stay, and functional capacity. CI=confidence interval; EMT=expiratory muscle training; MEP=maximal expiratory pressure; SD=standard deviation.

The effect of EMT compared to usual care was evaluated in two trials (n = 135)^[18,23]^. The pooled estimate showed that EMT improved expiratory muscle strength by 17.83 cmH₂O (95% CI: 78.59-27.07, *I^2^* = 0%) (Figure 3A). The overall quality of evidence according to the GRADE approach was rated as low quality (*i.e.*, downgraded for the risk of bias, imprecision, and publication bias) (Table 4).

**Table 4:** Evidence of expiratory muscle training compared to control group (minimal interventions) for expiratory muscle strength and functional capacity.

Certainty assessment							N. of patients		Effect	Certainty
N. of studies	Study design	Risk of bias	Inconsistency	Indirectness	Imprecision	Other considerations	Expiratory muscle training	Conventional physiotherapy	Absolute (95% CI)	
Maximal expiratory pressure (assessed with manovacuometry - cmH₂O)										
2	Randomized trials	Not serious	Not serious	Not serious	Serious^b^	Publication bias strongly suspected^c^	34	34	MD 19.31 cmH₂O higher (7.36 higher to 31.26 higher)	⨁⨁◯◯
										Low
Functional capacity (assessed with distance walked by means of the six-minute walk test - meters)										
2	Randomized trials	Not serious	Serious^a^	Not serious	Serious^b^	Publication bias strongly suspected^c^	34	34	MD 43.4 m higher (7.04 higher to 79.75 higher)	⨁◯◯◯
										Very low

CI=confidence interval; MD=mean difference

a Based on statistical criteria (*I^2^* > 50%) and the small number of published studies

b Based on a small number of events, we consider reduction of evidence, even with the seemingly narrow CI

c There are studies registered, but not published

#### Comparison Between Expiratory Muscle Training and Minimal Interventions for Functional Capacity

Of the six trials, two (n = 135) investigated the effect of EMT compared to usual care for functional capacity^[18,23]^. The pooled estimate showed that there was no difference between the studies about functional capacity (12.74 meters) (95% CI: -18.81-44.29, *I^2^* = 0%) (Figure 3B). The overall quality of evidence according to the GRADE approach was rated as very low quality (*i.e.*, downgraded for the risk of bias, inconsistency, imprecision, and publication bias) (Table 4).

### Sensitivity Analysis

Regarding sensitivity analysis, four comparisons exhibited heterogeneity > 40%. The comparison of IMT and minimal interventions for tidal volume, expiratory peak flow, and length of hospital stay had *I^2^* values of 81%, 54%, and 91%, respectively. The comparison of EMT and usual care for functional capacity had an *I^2^* value of 69%. Performing a sensitivity analysis was not possible because of the low number (two) of studies for each comparison. These heterogeneities are related to the clinical heterogeneity of the studies with different types of interventions and outcomes.

## DISCUSSION

RMT may significantly reduce complications induced by cardiac surgery. However, most studies are generally of low methodological quality, and they are highly heterogeneous with regard to the population, intervention, and measurement instruments investigated.

The current review meta-analysis focused on evaluating the effect of respiratory muscle strength training in the immediate postoperative period of cardiac surgery, especially in addition to strength, lung function, physical capacity, and length of hospital stay. Thus, the results demonstrated that the RMT improves both inspiratory and expiratory muscle strength^[12-15,23]^ and tidal volume^[13,14]^. Furthermore, no difference was found after RMT on the variables peak expiratory flow^[13,14]^, length of hospital stay^[13,15]^, and functional capacity^[18,23]^. These authors suggest that the low impact of RMT on these outcomes may be due to the small sample size and the number of losses that occurred in some studies, in addition to the lack of comparison between pre and postoperative values. In addition, there are still few clinical trials that have evaluated these outcomes, and future studies that do not present the previously described biases will be necessary to evaluate the effectiveness of the RMT on expiratory flow, length of hospital stay, and functional capacity.

Thus, to our knowledge, this was the first study that evaluated the efficacy of IMT and EMT in the immediate postoperative period of cardiac surgery based on the general quality of evidence, through GRADE.

Based on the GRADE approach, we also found that during the immediate postoperative period of cardiac surgery, the RMT was superior to usual care. Furthermore, the GRADE evidence for these results demonstrated a low to very low quality, without significative differences between studies that evaluated expiratory peak flow and length of hospital stay. In addition, based on very low evidence and no statistical difference, the EMT did not alter functional capacity. However, the results demonstrated that this therapy may improve expiratory muscle strength. In addition, IMT may improve inspiratory muscle strength, however, it is uncertain whether this therapy may improve tidal volume, peak expiratory flow, and functional capacity in this population.

Most of the clinical trials included in this review exhibited low methodological quality, according to PEDro^[19]^. This methodological analysis combined with GRADE^[16]^ allows better evaluation and grading of evidence and greater confidence in the results of the present study. A sensitive search strategy was used to identify the studies in the main databases. It was complemented by a manual search in the relevant studies and clinical trial registries. There were no language restrictions regarding the included studies, thus minimizing publication and language biases. However, it is possible that studies that were indexed only in local databases were missed and were consequently not included in this review. Only three studies registered on clinical trial registration platforms were tracked.

Since there is a risk of complications during the postoperative period of cardiac surgery, especially immediately after this procedure, the results of this review highlight the importance of respiratory strength training. As verified after this search, few studies have been developed evaluating expiratory strength training. Probably, since expiration is a passive mechanism which does not require as much force to be performed, studies have focused little on EMT. However, this fact does not justify not performing this exercise, since cardiac surgery reduces expiratory muscle strength, and maintaining it is essential for the good performance of lung function, as well as for maintaining ventilation and cough, which are essential for preventing infection, which may appear after surgery^[19,24,25]^.

In this review and meta-analysis, we demonstrated that EMT improves expiratory muscle strength, without present significative difference in the functional capacity, during the immediate postoperative period of cardiac surgery. However, only two trials used EMT for each outcome comparison. Additional studies should be carried out to better elucidate the effects of EMT immediately after cardiac surgery.

Pulmonary complications after cardiac surgery are directly related to the length of hospital stay, that is, the longer the hospital stay, the greater the risk of developing complications^[9]^. In this context, two eligible studies in this meta-analysis evaluated the effect of the RMT on this outcome. The study conducted by Cordeiro et al.^[15]^ demonstrated that IMT significantly reduced hospital stay days in patients undergoing CABG. And Barros et al.^[13]^ did find a difference in the length of hospital stay after this intervention.

IMT has been widely used in elective patients for cardiac surgery. However, most studies have investigated the effect of this intervention before cardiac surgery or after hospital discharge, during phases II or III of cardiac rehabilitation^[9-11]^.

Regarding IMT in the immediate postoperative period, our findings are similar to the ones of the review conducted by Gomes Neto et al.^[11]^. However, these authors did not associate EMT and did not use the GRADE approach to find a precise level of confidence in their results. Furthermore, they demonstrated an improvement of inspiratory muscle strength and pulmonary function, as well as a reduction of pulmonary complications, including trials performed up to June 2015, excluding recently published studies, which have been inserted in the present study.

The results of the present study indicate that IMT plays an important role in improving inspiratory muscle strength and tidal volume.

This finding shows the importance of RMT application in the acute postoperative period of cardiac surgery, which has reduced pulmonary function and inspiratory muscle strength, and consequently, complications.

### Limitations

There are some limitations that should be considered in this study. Although the search was very comprehensive, few studies were eligible for the application of this meta-analysis. Thus, these factors reduced sample size, associated with low methodological quality and few similar outcomes for the comparisons. In addition, some sex-based inferences should be considered, since most studies were conducted with men. The age of patients was also not specified in most studies. Furthermore, sensitivity analyses stratified by methodological quality were also not possible. Thus, only a small number of studies have been examined.

## CONCLUSION

In conclusion, this review demonstrated that both IMT and EMT demonstrated efficacy in improving respiratory muscle strength during the immediate postoperative period of cardiac surgery. There was no evidence indicating the efficacy of IMT for pulmonary function and length of hospital stay and the efficacy of EMT for functional capacity. Thus, we suggest that future studies should be performed to help elucidate the benefits of RMT in the immediate perioperative period of cardiac surgery.

**Table:** 

Authors’ Roles & Responsibilities	
TNA	Substantial contributions to the conception of the work; and the acquisition and analysis of data for the work; drafting the work; final approval of the version to be published
JPP	Substantial contributions to the acquisition and analysis of data for the work; drafting the work; final approval of the version to be published
EC	Substantial contributions to the acquisition of data for the work; final approval of the version to be published
EMC	Substantial contributions to the acquisition of data for the work; final approval of the version to be published
GG	Substantial contributions to the conception and design of the work; or the analysis of data for the work; drafting the work; final approval of the version to be published
